# How would Marie Kondo design a protocol? In favor of subtractive change in clinical trials

**DOI:** 10.3389/fmed.2026.1776929

**Published:** 2026-03-11

**Authors:** Rachel Bender Ignacio, Kathryn Rowan, Deborah Cook

**Affiliations:** 1 Division of Allergy and Infectious Diseases, Department of Medicine, University of Washington, Seattle, WA, United States; 2 Vaccine and Infectious Disease Division, Fred Hutchinson Cancer Center, Seattle, WA, United States; 3 Intensive Care National Audit & Research Centre, London, United Kingdom; 4 London School of Tropical Medicine and Hygiene, London, United Kingdom; 5 Department of Critical Care, St. Joseph's Healthcare Hamilton, Hamilton, ON, Canada; 6 Departments of Medicine, Health Research Methods, Evidence, and Impact, McMaster University, Hamilton, ON, Canada

**Keywords:** clinical trial, diversity and inclusion, protocol design and implementation, regulatory science, research methods

Complexities in contemporary clinical trials are frequently self-defeating relative to the goals of clinical research—namely to inform and improve care for as many patients as possible. Trials need to be rigorous, relevant, ethical, inclusive, feasible and affordable. Trials require broad access to research participation to optimize the generalizability of results and influence practice and improve outcomes for diverse populations. The degree of complexity of many clinical trials today too often restricts their conduct to academic centers or specialized research organizations where only sufficiently resourced investigators can conduct them, narrowing the populations of participants having access to trials. Limiting who conducts, delivers, and participates in trials decreases the representation of participants and threatens the generalizability of trial results to under-represented persons. Both internal and external validity may be negatively affected.

Unnecessary complexity risks recruitment and retention challenges, slows time to trial completion, and requires additional research resources, driving up costs often with unclear benefit. Delayed knowledge acquisition creates lags in evidence generation and application in practice. The benefits of health care advances are particularly slow to reach marginalized populations and those in low- and middle-income countries. Experiences during the COVID-19 pandemic provided a window into ways that trials could become simpler and more accessible. However, much of the regulatory guidance that allowed these adjustments has reverted to “business as usual”, foregoing these useful simplifications.

We outline how the trials enterprise has lost the proportionality that should inform trial complexity. Humans consistently default to additive changes and are less likely to identify opportunities for subtractive change—that is, removing components and complexity (1). However, the “more is more” approach runs counter to our ethical imperatives to represent relevant, diverse populations in our research, limit participant burdens, and to efficiently and economically generate impactful results. Although leading trialists have been calling for the design of large, simple trials for decades, the trend has been toward more complex procedures, rather than simplicity since then (2). Large, simple trials are an obvious starting point for the issues we highlight here. Proportional simplicity requires retaining more stringent approaches for trials that are “high risk” (first-in-human, vulnerable populations, high-risk interventions (e.g. chimeric antigen receptor T cells)), registrational trials, or those focused on mechanistic exploration. However, even the complexity of these “higher risk” trials needs careful review and reconsideration.

## How did trials become so complex?

The creep in trial complexity is multifactorial. Necessary protections have been added to address structural research harms that have disproportionately impacted vulnerable populations, including Black and Indigenous communities and incarcerated people, and populations in formerly colonized countries. Complexities were introduced to satisfy regulatory authorities (Food and Drug Administration (FDA), European Medicines Agency (EMA), etc.) for new drugs and devices, but are often applied across the board, including in trials of lower-risk and post-approval interventions. A vicious cycle of exhaustive data collection and monitoring, and query generation and feedback, can penalize lack of exact adherence to processes for research personnel and adds unnecessary burden for participants. This “regulatory-industrial complex” (3) is enforced by contract research organizations (CROs)—generally for-profit companies—whose role is to execute and/or monitor trials on behalf of sponsors. There is a tipping point at which the unintended consequences of complexity jeopardize trial feasibility and affordability, especially as future research funding is likely to be further constricted in many jurisdictions.

## Maintaining ethical integrity, decreasing research waste

Returning to the premise that trials should be rigorous, relevant, ethical, inclusive, feasible and affordable, means that judicious *subtractive* change must come from funders and regulators but involve all impacted parties, especially affected communities (strategies in Table 1). Trial simplification could initially focus on late-phase and post-approval studies, but be addressed for most designs, retaining more stringent approaches for trials that are “high risk”.

**Table 1:** Strategies for reducing complexity and burden in the design and delivery of clinical trials.

**Suggested Strategies**	**Relevant parties**
**Streamline regulatory processes and eligibility**	
• Simplify inclusion criteria, when possible, to enhance eligibility	Investigators, FDA/EMA
• Focus exclusion criteria on key safety or ethical concerns to avoid very narrow participant representation	Investigators, FDA/EMA
• Develop reciprocal regulatory and ethics review to minimize duplication; track turn-around times to address delays. Ensure that the Single IRB approach avoids duplicating local processes which have not been eliminated	Office of Human Research Protection, other national ethics offices
**Rationalize informed consent procedures**	
• Shorten and simplify informed consent forms and templates	Investigators, Sponsors, IRBs/ECs
• Use reconsenting procedures only when the participants' status, risk or procedural burden is changed	Sponsors, IRBs/ECs
• Allow participant education or notifications instead of reconsent when study updates would not impact safety or willingness to continue on study	Sponsors, IRBs/ECs, CROs
• Allow non-study-specific, short-form consents in many languages that facilitate the use of verbal translation	IRBs/ECs, national research protection offices
**Allow authorized/certified clinical testing, sites, and data sources**	
• Relax the practice of requiring all sites of research conduct to be pre-specified and validated separately for research (i.e., specified on an FDA form 1572)	FDA/EMA, Sponsors
• Permit research follow-up opportunities in external clinics outside the main research site	FDA/EMA, Sponsors
• Allow safety monitoring and clinical endpoints from local laboratory or imaging reports that are documented rather than study-collected	FDA/EMA, Sponsors
**Liberalize restrictions that prevent “out of the clinic” protocol implementation**	
• Incorporate phrases such as “hybrid” or “remote” in the protocol to allow flexibility in location of research performance	Investigators, Sponsors, Monitors/CROs
• Incorporate TASSO™ or other remote blood draws, self-swab collection	Sponsors, FDA/EMA
• Permit home monitoring with wearables, video-assisted gait assessment etc for safety and outcome assessment	Sponsors, FDA/EMA
**Decrease data collection burden for researchers**	
• Review and trim case report forms to include only data that realistically will be used	Sponsors, Investigators
• Allow for collection of data already embedded in the clinical record	Sponsors, FDA/EMA
• Collect advanced biological samples (e.g., PBMCs) only in centers which can efficiently, cost-effectively collect, store and submit them	Sponsors, Investigators
**Decrease data collection burden for participants**	
• Consider a hierarchy of procedures for abbreviated trial visits if necessary	Sponsors, Monitors/CROs
• Minimize questionnaires to reflect the trial outcomes; consider short form versions; make some forms optional for those without English proficiency; translate key forms	Sponsors, Investigators
• Obtain patient/community feedback; pilot processes before starting	Sponsors, Investigators
**Develop flexible protocols to allow site-specific sub-study participation**	
• Consider which sub-studies or exploratory endpoints are truly needed for safety and scientific integrity	Sponsors, Investigators
• Consider making sub-studies optional for sites with capacity, and for participants with fewer barriers to participation	Sponsors, Investigators
**Rationalize data monitoring**	
• Align monitoring intensity with the overall study risk: alignment with ICH E6(R3)	Sponsors, CROs, FDA/EMA
• Focus monitoring on ensuring safety and rigor, not on finding errors	Sponsors, CROs
• Adapt later trial monitoring strategies to early trial findings	Sponsors, CROs
• Remove reporting and site penalization for minor errors that do not impact research integrity, such as transcription errors or participant errors (ie. missed doses, diaries)	Sponsors, CROs, FDA, EMA
• Remove multiple layers of monitoring, response to monitoring, and documentation of retraining	Sponsors, CROs, FDA/EMA
• Amend data safety monitoring reports for brevity to improve timeliness and effort	Sponsors, CROs, FDA/EMA
**Reduce redundancy in training and systems**	
• Consolidate the number of unique data entry and monitoring systems, and validations on those external systems	Sponsors, Investigators, FDA/EMA
• Reduce the training and onboarding requirements for each study to improve feasibility	Sponsors, CROs
**Rationalize adverse event reporting**	
• Remove expected study outcomes from safety reporting obligations to avoid duplication (eg: myocardial infarction in a cardiovascular prevention study)	Sponsors, Investigators
• Reduce reporting requirements for adverse events when interventions are low risk or when they are unrelated to the intervention, especially for implementation trials	Sponsors, Investigators, FDA/EMA, Research Ethics Offices
**Decrease the barrier to entry for investigators/research teams**	
• Embed mentorship and research training in clinical and public health training	Academic institutions, Investigators, National Health Systems
• Offer real-world training for early career investigators	Academic and Medical Education programs, Investigators
• Allow clinical pharmacies to prepare study products under usual guidelines especially if not blinded; use clinical supply for approved drugs	FDA/EMA, Sponsors
• Support research readiness initiatives to maintain infrastructure such that each new study can launch more quickly	Academic and Public Health Institutions, Investigators, National Health Services systems (HHS, NHS etc)

FDA, US Food and Drug Administration; EMA, European Medicines Agency; IRBs, Intitutional Review Boards; ECs, Ethics Committees; PBMC, peripheral blood mononuclear cells; CRO, contract research organization; HHS, US Department of Health and Human Services; NHS, United Kingdom National Health Service.

The 2025 E6(R3) revision to The International Council on Harmonization (ICH) Guideline for Good Clinical Practice (4) emphasizes proportionality in trials. If implemented correctly, monitoring proportionality increases flexibility of trial designs while retaining focus on participant safety and data accuracy.

## Simplicity and flexibility

Decreasing the stringency of trial procedures and processes could allow for greater participation of community and research-naïve sites, and lead to inclusion of participants outside of major medical centers. Revisions to ICH decrease emphasis on perfection, instead favoring innovations that allow flexibility to bring trials to people via remote or hybrid designs, and enable patient-collected data. Regulatory and ethics authorities, such as the FDA and EMA, national offices of human research protections, sponsors, and monitors should begin, where feasible, to integrate clinically-obtained data, allowing measurements from self-performed home vital sign monitoring, wearable physiologic devices, and blood self-collection kits.

Mechanistic substudies or more complex procedures, including collection and banking of samples that require specialized processing or cryopreservation, could be limited to selected, well-resourced research sites and be optional for other sites. Protocols should allow for accredited, non-research laboratories or other standard-of-care procedures to report data without extensive re-validation. Allowing procedures to be performed locally, or remotely, or with more flexible hours facilitates participation by those with shift-work jobs, those who require childcare coverage (a common reason women underenroll in research (5)), or those who live outside metropolitan areas.

Lengthy informed consent documents, often further complicated by each institutions' risk management and legal departments, can create a façade of integrity with no face validity. Lengthy consent documents may deter potential participants and discriminate against others, particularly those with low (health) literacy or earned distrust in the healthcare system. Re-consenting participants for administrative changes that do not impact risk should be minimized, as reconsenting may only increase participant burden and jeopardize their retention. One way to include impacted communities and decrease the research burden is to include Community Advisory Board (CAB) feedback on study design, with special focus on the informed consent documents and schedule of activities (6, 7). More than requiring that the informed consent document meets a specified reading level, community imput can make sure that the document places appropriate emphasis on the components that are the most salient in their view after learning about the proposed intervention in the context of their disease (8). This feedback will also inform whether potential participants find the planned research activities feasible (e.g., regarding number of questionnaires) and affordable (e.g., personal travel to research centers in follow-up). Clear, simple informed consent processes must extend beyond the written informed consent document to facilitate participation by people who speak various languages, considering synchronous medical translation services, visual abstracts, or video-assisted informed consent encounters to illustrate trial processes (9).

Extensive trial eligibility criteria should be simplified to enroll the broadest participant population that maintains safety and responsiveness to the research question. Trials attempting to evaluate a “clean” population with only the disease under study will naturally exclude older people and those from marginalized backgrounds who are more likely to have accumulated comorbidities. Women, children, the elderly, and those with common medical conditions are frequently excluded from multicenter trials, which should be transparently justified in protocols, or better yet, reconsidered (10).

The introduction of a single institutional review board has the potential to simplify and streamline ethics review. This has been realized in many situations, but requires tracking to be sure that an approach doesn't duplicate work or increase the regulatory and reporting requirements to accommodate all parties. The US National Cancer Institute launched their Central Institutional Review Board (CIRB) in 2001, and has demonstrated time savings and faster approvals in regulatory submissions made by partner institutions. One report demonstrated net cost-savings, except that as used at the time of this study, many institutions were not fully delegating review to CIRB, and this analysis was limited in studying only time for local approval after the study was centrally approved (11).

Intensity of data collection should mirror the trial's aim. For some trials, a very small proportion of data collected on lengthy case report forms is ever used, creating waste and opportunity cost (12). Further, data is increasingly being leveraged from electronic health records and registries for trials. For example, the TASTE study, which evaluated thrombus aspiration during ST-elevation myocardial infarctions, embedded study screening into clinical practice, relied on clinical data, and cost a fraction of most trials (13). Advances in electronic health record interoperability and data standardization could support re-use of health data for multiple purposes, and augment clinical trial data infrastructure in the future (14).

Adverse Event (AE) reporting, similarly, can be contextualized to avoid wasting resources on detailed documention that has no impact on either the trial's internal validity or participant safety. For trials testing interventions already in clinical practice, events that are possible consequences of that intervention could be pre-determined as trial outcomes rather than AEs; events that are part of the natural history of a disease process should similarly not trigger AEs (15).

The scope and frequency of data monitoring should be proportionate to risk and adapted to early trial findings. One approach, supported by ICH E6(R3), is “risk-based monitoring”, whereby intensive monitoring is reserved for ethics assurances (eg. informed consent), outcomes that would change study results if missing or erroneous, and key safety data. The over-used label “pragmatic trial” is not a synonym for casually designed or implemented; proportional but robust approaches to monitor key details are also critical for pragmatic trials.

Changes to data collection and data monitoring will require concerted collaborative efforts from regulatory authorities and sponsors, including direction to CROs and investigators to focus on subtractive change and avoid “one size fits all” approaches (16).

## Conclusions

The pandemic underscored the vital role of research for society, highlighting the need to optimize and streamline procedures and processes, while maintaining ethics and rigor. Some trials for emerging infections innovated within this broken research infrastructure, (17) but the present maximalist approach continues to challenge principals of making research accessible, equitable, timely, inclusive, and applicable (18). *What if Marie Kondo wrote a protocol?* We must carefully consider which parts of clinical trials *do* serve participants and uphold research integrity, and which are needless clutter. The art of subtractive change, popularized by Kondo (16) and long-championed in the Toyota Way of continuous improvement (19), can be applied to clinical trials. Subtractive change can facilitate our collective aims without exhausting the limited resources of the research enterprise, the most important of which is our study participants.
