# Correction: How would Marie Kondo design a protocol? In favor of subtractive change in clinical trials

**DOI:** 10.3389/fmed.2026.1838161

**Published:** 2026-04-07

**Authors:** Rachel Bender Ignacio, Kathryn Rowan, Deborah Cook

**Affiliations:** 1 Division of Allergy and Infectious Diseases, Department of Medicine, University of Washington, Seattle, WA, United States; 2 Vaccine and Infectious Disease Division, Fred Hutchinson Cancer Center, Seattle, WA, United States; 3 Intensive Care National Audit & Research Centre, London, United Kingdom; 4 London School of Tropical Medicine and Hygiene, London, United Kingdom; 5 Department of Critical Care, St Joseph's Healthcare Hamilton, Hamilton, ON, Canada; 6 Departments of Medicine, Health Research Methods, Evidence, and Impact, McMaster University, Hamilton, ON, Canada

**Keywords:** clinical trial, diversity and inclusion, protocol design and implementation, regulatory science, research methods

The title of this article was erroneously given as: “How would Mari Kondo design a protocol? In favor of subtractive change in clinical trials”. The correct title of the article is “How would Marie Kondo design a protocol? In favor of subtractive change in clinical trials”.

The original version of this article has been updated.

